# Correction to “Loss of Consciousness in a Child Following Accidental Ingestion of Brimonidine Ointment: A Case Report”

**DOI:** 10.1002/ccr3.73072

**Published:** 2026-07-01

R. Zare Mahmoudabadi, M. Vafapour, M. Karubi, et al., “Loss of Consciousness in a Child Following Accidental Ingestion of Brimonidine Ointment: A Case Report,” *Clinical Case Reports* 14, no. 6 (2026): e72901, https://doi.org/10.1002/ccr3.72901.

In the originally published version of this article, the names of the first and third authors were incorrectly published. The first author's name was published as “Ramin Zareh Mahmoudabadi” and should be corrected to “Ramin Zare Mahmoudabadi”. The third author's name was published as “Majid Karrobi” and should be corrected to “Majid Karubi”.

The online version of this article has been corrected accordingly.

We apologize for this error.

